# Pulmonary Arterial Hypertension in Adults: Novel Drugs and Catheter Ablation Techniques Show Promise? Systematic Review on Pharmacotherapy and Interventional Strategies

**DOI:** 10.1155/2014/743868

**Published:** 2014-06-12

**Authors:** Salvatore Rosanio, Francesco Pelliccia, Carlo Gaudio, Cesare Greco, Abdul M. Keylani, Darrin C. D'Agostino

**Affiliations:** ^1^Division of Cardiology, Department of Internal Medicine, University of North Texas Health Science Center, 855 Montgomery Street, PCC Room 315, Fort Worth, TX 76107, USA; ^2^Department of Heart and Great Vessels “Attilio Reale”, La Sapienza University, Rome, Italy

## Abstract

This systematic review aims to provide an update on pharmacological and interventional strategies for the treatment of pulmonary arterial hypertension in adults. Currently US Food and Drug Administration approved drugs including prostanoids, endothelin-receptor antagonists, phosphodiesterase type-5 inhibitors, and soluble guanylate-cyclase stimulators. These agents have transformed the prognosis for pulmonary arterial hypertension patients from symptomatic improvements in exercise tolerance ten years ago to delayed disease progression today. On the other hand, percutaneous balloon atrioseptostomy by using radiofrequency perforation, cutting balloon dilatation, or insertion of butterfly stents and pulmonary artery catheter-based denervation, both associated with very low rate of major complications and death, should be considered in combination with specific drugs at an earlier stage rather than late in the progression of pulmonary arterial hypertension and before the occurrence of overt right-sided heart failure.

## 1. Introduction

Pulmonary arterial hypertension (PAH) is a progressive, fatal syndrome characterized by increased pulmonary vascular resistance that leads to right-sided heart failure and, eventually, death [1]. The incidence and prevalence of PAH are estimated at 2.4–7.6 cases/million/yr and 15–26 cases/million/yr, respectively, in large population studies with ~2 : 1 female-male ratio [2, 3].

Contemporary one-, three-, five-, and seven-year survival rates from time of diagnostic right-sided heart catheterization are 85%, 68%, 57%, and 49%, respectively [4].

By expert consensus, PAH is regarded as mean pulmonary artery pressure >25 mmHg, pulmonary vascular resistance >3 Wood units, pulmonary capillary wedge pressure <15 mmHg, and normal or reduced cardiac output in absence of other causes of pulmonary hypertension [5].

Based on the World Health Organization (WHO) classification, PAH comprises different forms (WHO Group 1): idiopathic, heritable PAH (due to* bone morphogenetic protein receptor type 2, activin receptor-like kinase-1, endoglin, decapentaplegic 9*,* caveolin-1*, or* KCNK3* gene mutations), anorexigen-induced PAH, and medical conditions associated with PAH (including portal hypertension, connective tissue disease [most commonly systemic sclerosis], human immunodeficiency virus, schistosomiasis, chronic hemolytic anemia, and congenital heart disease) [6].

Besides WHO Group 1 PAH, other forms of pulmonary hypertension include WHO Groups 2 (pulmonary venous hypertension), 3 (pulmonary hypertension due to hypoxemia), 4 (chronic thromboembolic pulmonary hypertension), and 5 (miscellaneous or multifactorial) [6].

Vasoconstriction, proliferative, and obstructive remodeling of the pulmonary vessel wall, inflammation, apoptosis resistance, plexiform lesions, and thrombosis* in situ* contribute to increased pulmonary vascular resistance in PAH [7–11]. Genetic and pathophysiologic studies have emphasized the relevance of a number of mediators in this condition, including prostaglandin I_2_ (prostacyclin), endothelin-1, nitric oxide, angiopoietin-1, serotonin, cytokines, chemokines, and members of the transforming-growth factor-beta superfamily [11]. Thus, these molecules represent logical pharmacological targets.

On the other hand, animal and clinical studies demonstrated an increased sympathetic activity in PAH [12–17]. Of note, it has been shown that distension of the main pulmonary artery reflexly (via sympathetic nerves) causes a significant rise in pulmonary vascular resistance by excitation of baroreceptors in or near the bifurcation of the main pulmonary artery [12–17]. Hence, denervation of the pulmonary vasculature is a reasonable therapeutic target.

As authors of the present paper and practicing cardiologists, we see patients with pulmonary hypertension on a regular basis. Although this is most commonly in the form of pulmonary venous hypertension related to elevated left heart pressures (WHO Group 2), the remarkable advances within the last 5 years in our understanding of the epidemiology, pathogenesis, and pathophysiology of PAH compel cardiologists to be more acquainted of this devastating disease.

In this review, we summarize the mechanism of action, clinical data, and regulatory histories of US Food and Drug Administration (FDA) approved drugs for PAH and we discuss as well the latest development of novel compounds and future targets for therapeutics, including interventional strategies such as the promising percutaneous radiofrequency catheter-based pulmonary artery denervation.

## 2. Pharmacotherapy

Multiple randomized controlled trials have been performed in PAH resulting in the regulatory FDA approval of nine drugs of four pharmacological classes: prostanoids, endothelin-receptor antagonists, phosphodiesterase type-5 inhibitors, and guanylate-cyclase stimulators.

### 2.1. Prostanoids

Prostacyclin, the main product of arachidonic acid in the vascular endothelium, induces relaxation of vascular smooth muscle by stimulating the production of cyclic-adenosine monophosphate and inhibits the growth of smooth-muscle cells [10, 18, 19]. In addition, this molecule is the most potent endogenous inhibitor of platelet aggregation. Dysregulation of the prostacyclin metabolic pathways has been shown in patients with PAH. Studies of excreted prostacyclin metabolite levels and prostacyclin synthase expression in lung tissue indicate that prostacyclin synthesis is reduced in patients with PAH compared with healthy controls, providing a rationale for treating PAH with synthetic prostacyclin analogues (prostanoids) [10, 18, 19].

The clinical effects of approved prostanoids (namely, epoprostenol, iloprost, and treprostinil) have been tested in several randomized controlled clinical trials, which are summarized in Table 1.

### 2.2. Epoprostenol

It has a very short half-life (3–6 min) and limited stable time at room temperature (<8 hours). It requires to be continuously administered by an infusion pump or a permanent indwelling catheter. The efficacy of epoprostenol has been tested in three unblinded randomized controlled trials in idiopathic/heritable PAH and PAH associated with systemic sclerosis (Table 1) [20–22]. This agent improves symptoms, exercise capacity, and hemodynamics in both clinical conditions; however, increased survival rate was only observed in idiopathic PAH. In the study by Barst et al. in 81 adults with idiopathic PAH and functional class III (moderate) or IV (severe) symptoms, despite optimal medical therapy, 8 of the 40 patients (20%) receiving conventional therapy alone died at the end of the 12-week treatment period, whereas none of the 41 patients receiving epoprostenol died (*P* = 0.003) [21]. Although the trial was not fully blinded, it remains the only randomized, controlled trial to show a survival benefit in patients with PAH.

The FDA approved epoprostenol in 1995 for PAH patients with WHO functional classes III and IV who do not respond adequately to conventional therapy such as diuretics, oral anticoagulants, and long-term oxygen and digoxin or calcium-channel blockers when indicated. It is unusual for a patient to be awaiting lung transplantation without receiving epoprostenol.

Subsequent label revisions have included the addition of patients with PAH related to systemic sclerosis/scleroderma (year 2000) and all patients with PAH regardless of etiology to improve exercise capacity (year 2011).

Treatment with epoprostenol is initiated at a dose of 2–4 ng/kg/min, with doses increasing at a rate limited by side effects (flushing, headache, diarrhea, and jaw or leg pain). The optimal dose varies between individual patients, ranging between 20 and 40 ng/kg/min.

Serious adverse events related to the delivery system include pump malfunction, local site infection, catheter obstruction, and sepsis. Additionally, abrupt interruption of the infusion should be avoided as, in some patients, this may lead to a rebound pulmonary hypertension with symptomatic deterioration and even death.

### 2.3. Iloprost

It has a serum half-life from 20 to 25 min. The pulmonary vasodilating effects of inhaled iloprost last nearly 45 min; therefore, 6 to 9 daily inhalations of 2.5 *μ*g or 5.0 *μ*g are needed, with each of them requiring ~30 min.

Its regulatory FDA approval occurred in 2004 and was based on the results from one pivotal multicenter trial, AIR (Aerosolized Iloprost Randomized) [23].

The trial was conducted in Europe and enrolled 203 adult patients with moderate or severe inoperable chronic thromboembolic pulmonary hypertension (WHO Group 4) or PAH that was idiopathic or associated with scleroderma or appetite-suppressant drugs. The primary end point was a combined measure of improvement of at least one functional class and at least 10% in 6 min walk distance from baseline to week 12 (Table 1).

The end point was reached by 17% of patients receiving iloprost and 5% of those receiving placebo (*P* < 0.05). The study also showed an improvement in pulmonary vascular resistance and clinical events in patients treated with iloprost.

### 2.4. Treprostinil

It has a terminal elimination half-life of ~2–4 hours and is administered either by inhalation, by a microinfusion pump for continuous subcutaneous infusion, by a pump for continuous intravenous infusion, or orally.

Treprostinil was approved by the FDA in 2002 for continuous subcutaneous infusion on the basis of a 12-week, multicenter, randomized, double-blind, placebo-controlled trial.

Simonneau et al. evaluated 470 adults with functional class II to class IV status and PAH that was idiopathic, related to connective tissue disease, or related to congenital systemic-to-pulmonary shunts (Table 1) [24].

Patients treated with treprostinil increased 6 min walk distance and had better quality of life and improved pulmonary hemodynamics and symptoms.

Infusion site pain and reactions are the most common adverse events with subcutaneous treprostinil; these events are reported in more than 80% of patients but wane over time many of them.

In 2004, on the basis of data establishing bioequivalence, the FDA approved an intravenous formulation of treprostinil for patients with PAH in functional classes II to IV who do not tolerate the subcutaneous form or in whom intravenous administration may be preferable to subcutaneous infusion providing at the same time the advantage of less frequent need for drug reservoir replacement.

In 2009 inhaled treprostinil was licensed by the FDA to improve functional capacity in patients with PAH in functional class III, with recommended four times daily dosing.

The approval was based on the results from the TRIUMPH-1 (TReprostinil Sodium Inhalation Used in the Management of Pulmonary Arterial Hypertension) trial (Table 1) [25].

Two hundred thirty-five PAH patients with functional class III (98%) or IV symptoms and a 6 min walk distance from 200 to 450 m while being treated with bosentan (70%) or sildenafil were randomized to inhale treprostinil (up to 54 *μ*g) or inhale placebo 4 times daily. The primary end point was peak 6 min walk distance at 12 weeks. Secondary end points included time for clinical worsening, Borg dyspnea score, functional class, 12-week trough 6 min walk distance, 6-week peak 6 min walk distance, quality of life, and PAH signs and symptoms. The Hodges-Lehmann between-treatment median difference in change from baseline in peak 6 min walk distance was 19 m at week 6 (*P* = 0.0001) and 20 m at week 12 (*P* = 0.0004). Quality of life measures improved on active therapy. There were no improvements in the other secondary end points.

Inhaled treprostinil was safe and well-tolerated. The most common adverse events, occurring in ≥10% of treprostinil-treated patients, were cough, headache, throat irritation, or pharyngolaryngeal pain.

In December 2013, the FDA licensed treprostinil extended-release oral tablets for the treatment of PAH to improve exercise capacity. This approval marks the first time that the FDA has approved an orally administered prostanoid for any disease.

The primary efficacy trial, FREEDOM-M (Oral Treprostinil as Monotherapy for the Treatment of Pulmonary Arterial Hypertension), was a double-blind, randomized, placebo-controlled, parallel group study comparing the twice-daily administration of oral treprostinil to placebo in* de novo* PAH (Table 1) [26]. Three hundred forty-nine patients (intent-to-treat population) not receiving endothelin-receptor antagonist or phosphodiesterase type-5 inhibitor background therapy were randomized with access to 0.25 mg treprostinil tablets at randomization. The primary end point was a change from baseline in 6 min walk distance at week 12. Secondary end points included Borg dyspnea index, clinical worsening, and symptoms of PAH. The week 12 treatment effect for 6 min walk distance (modified intent-to-treat population) was 23 m (*P* = 0.0125). For the intent-to-treat population, 6 min walk distance improvements were observed at peak (26 m; *P* = 0.0001) and trough (17 m; *P* = 0.0025) plasma study drug concentrations.

Treprostinil was well tolerated and side effects were typical of prostacyclin treatments. In contrast to prior clinical trials, there was no significant treatment effect on the incidence of clinical worsening. However, the overall incidence of clinical worsening was quite low.

Oral treprostinil could provide a convenient, first-line prostacyclin treatment option for PAH patients not requiring more intensive therapy.

## 3. Endothelin-Receptor Antagonists

The endothelin system has a major role in the pathogenesis of PAH. Activation of the endothelin system has been shown in both plasma and lung tissue of PAH patients [9]. Although it is still unknown if the increases in endothelin-1 (ET-1) plasma levels are a cause or a consequence of PAH [36, 37].

The biological effects of ET-1 are regulated primarily by two distinct receptors, ET_A_ and ET_B_. Activation of ET_A_ receptors causes sustained vasoconstriction and proliferation of vascular smooth-muscle cells, whereas ET_B_ receptors mediate pulmonary endothelin clearance and induce the production of nitric oxide and prostacyclin by endothelial cells that may counterbalance the deleterious effects of ET-1 [36, 37].

Despite potential differences in receptor isoform activity, the efficacy in PAH of dual ET_A_/ET_B_ receptor antagonist drugs and of selective ET_A_ blockers appears to be comparable.

Currently, there are three ET-1 receptor antagonists as first-line use in patients with mild to moderate PAH: bosentan, ambrisentan, and macitentan.

Table 2 summarizes the clinical characteristics of patients, etiology, end points, treatment effects, and adverse reactions in the pivotal Phase III randomized controlled trials, which determined the approval of these agents [27–31].

### 3.1. Bosentan

It is an oral active dual ET_A/B_ receptor antagonist and its approval in 2001 was based on two randomized, double-blind, placebo-controlled trials.

The initial pilot trial by Channick et al. evaluated 32 patients with PAH (idiopathic or associated with connective tissue disease) and functional class III symptoms (Table 2) [27]. Patients were randomized to receive either bosentan (62.5 mg taken twice daily for 4 weeks and then 125 mg twice daily) or placebo for a minimum of 12 weeks. Bosentan significantly improved 6 min walk distance change from baseline to week 12 (mean placebo-adjusted change, +76 m; *P* = 0.02); this improvement was maintained through week 20. Bosentan resulted also in a statistically significant improvement in pulmonary arterial pressure (mean change from baseline for bosentan, −1.6 mmHg; placebo, +5.1 mmHg; *P* = 0.01), cardiac index, and pulmonary vascular resistance. This agent significantly improved functional class status and time to clinical worsening compared with placebo.

The multicenter BREATHE-1 (Bosentan Randomized Trial of Endothelin Antagonist THErapy) trial evaluated 213 patients with PAH (idiopathic or associated with connective tissue disease) and functional class III or IV (Table 2) [28]. Subjects were randomized to receive bosentan 62.5 mg twice daily for 4 weeks followed by either 125 or 250 mg twice daily or placebo for 16 weeks. Pooled bosentan data for both doses showed a 44 m treatment effect for the primary efficacy end point of change in mean 6 min walk distance from baseline to week 16. When data were evaluated by each dose group, the (placebo-adjusted) change in 6 min walk distance showed a difference between doses: +35 m with 125 mg and +54 m with 250 mg. Bosentan also significantly improved functional class and reduced the time to clinical worsening at week 16 defined as death, lung transplantation, hospitalization for PAH, and no improvement or worsening leading to discontinuation, need for epoprostenol therapy, or atrial septostomy. Although the difference between each bosentan arm and controls for time to clinical worsening was significant at both weeks 16 and 28, the differences were the highest for the patients followed through week 28.

Elevated liver aminotransferase values >3 times normal occurred in ~13% of patients receiving bosentan, with a higher incidence in the group receiving the 250 mg dose. However, there were no reports of jaundice or liver failure. Despite a greater efficacy with 250 mg dose, the increased risk of hepatotoxicity resulted in FDA approval of the lower dose of 125 mg. In addition, FDA approval required patients to obtain liver function tests at least monthly through a restricted drug distribution program with either dose reduction, interruption of treatment, or permanent discontinuation depending upon aminotransferase values. Testing for pregnancy is also required monthly in women of childbearing potential.

The most common adverse events observed with bosentan treatment are headache, flushing, and syncope. Reductions in hemoglobin levels and impaired spermatogenesis have also been observed.

In 2009, the FDA expanded the bosentan label for patients with functional class II on the basis of the EARLY (Endothelin Antagonist tRial in mildLY symptomatic pulmonary arterial hypertension patients) [29]. This study evaluated exercise capacity and hemodynamics (pulmonary vascular resistance) in 185 patients with WHO functional class II (16% receiving stable doses of sildenafil) who were treated for 6 months (Table 2). At month 6, mean pulmonary vascular resistance was 83% of the baseline value in the bosentan group and 107% of the baseline value in the placebo group (treatment effect 23%; *P* < 0.0001). The favorable effect of bosentan on pulmonary vascular resistance was also confirmed in the subgroup of patients treated with sildenafil. Mean 6 min walk distance increased from baseline in the bosentan group (+11 m) and decreased in the placebo group (−8 m), with a mean treatment effect of 19 m (*P* = 0.07).

### 3.2. Ambrisentan

It is an oral selective ET_A_-receptor antagonist (ET_A_ *versus *ET_B_ receptor > 4000-fold) with a bioavailability and half-life that allow once daily dosing. In 2007, the FDA approved 5 and 10 mg ambrisentan for the once daily treatment of patients with PAH and functional class II or III symptoms to improve exercise capacity and delay clinical worsening. Its approval was based on ARIES-1 and ARIES-2 (Ambrisentan in pulmonary arterial hypertension, RandomIzed, double-blind, placebo-controlled, multicenter, Efficacy Study) trials (Table 2) [30].

Both trials were identical in design (except for overlapping doses) and conducted in different countries. ARIES-1 (US, Mexico, South America, Australia, and Europe) enrolled 202 patients who received 5 or 10 mg of ambrisentan or placebo for 12 weeks. ARIES-2 (Europe, South America, and Israel) enrolled 192 patients who received 2.5 or 5 mg of ambrisentan or placebo for 12 weeks. The 6 min walk distance (primary end point) increased in all ambrisentan arms; mean placebo-corrected treatment effects were 31 m (*P* = 0.008) and 51 m (*P* < 0.001) in ARIES-1, respectively, and 32 m (*P* = 0.022) and 59 m (*P* < 0.001) in ARIES-2, respectively.

Improvements in time to clinical worsening (ARIES-2), WHO functional class (ARIES-1), several SF-36 Health Survey subscales (quality of life; ARIES-2), Borg dyspnea score (both studies), and B-type natriuretic peptide (both studies) were observed. In 280 patients completing 48 weeks of treatment with ambrisentan monotherapy, the improvement from baseline in 6 min walk distance at 48 weeks was 39 m.

Ambrisentan was well tolerated in both trials, with headache being the most frequent adverse event. No patient treated with ambrisentan developed aminotransferase concentrations >3 times the upper limit of normal.

In 2011, the FDA removed the warning label for liver injury and requirement for monthly liver function testing for ambrisentan on the basis of postmarketing data involving more than 7,800 patient years.

### 3.3. Macitentan

This dual ET_A/B_ receptor antagonist was developed by modifying the structure of bosentan to increase efficacy and safety. Macitentan is characterized by sustained receptor binding and enhanced tissue penetration [31, 38].

In October 2013, the FDA licensed 10 mg macitentan for the once daily treatment of patients with PAH and functional class II or III symptoms to delay disease progression. The approval was based on results from SERAPHIN (Study with an Endothelin Receptor Antagonist in Pulmonary Arterial Hypertension to Improve cliNical Outcome), a double-blind, placebo-controlled, Phase III trial (Table 2) [31].

This event-driven study, conducted in 151 centers from almost 40 countries in North and Latin America, Europe, Asia-Pacific, and Africa, randomized 250 patients to placebo, 250 to the 3 mg macitentan dose, and 242 to the 10 mg macitentan dose. Mean follow-up period was 85 weeks for those assigned placebo, 99 weeks for those assigned 3 mg macitentan, and 104 weeks for those assigned 10 mg macitentan.

The primary end point was the time from the initiation of treatment to the first occurrence of a composite end point of death, atrioseptostomy, lung transplantation, initiation of treatment with intravenous or subcutaneous prostanoids, or worsening of PAH. The end point was reached by 46%, 38%, and 31% of the patients in these groups, respectively. The hazard ratio (HR) for the 3 mg macitentan dose* versus* placebo was 0.70 (97.5% confidence interval [CI], 0.52–0.96; *P* = 0.01), and the HR for the 10 mg macitentan dose was 0.55 (97.5%CI, 0.39–0.76; *P* < 0.001). Worsening of PAH was the most frequent event. Death or hospitalization due to PAH occurred in 34% of the patients in the placebo group, 26% of those in the 3 mg macitentan group, and 21% of those in the 10 mg macitentan group. The HR for a 3 mg daily dose of macitentan versus placebo was 0.67 (95%CI, 0.46–0.97; *P* = 0.01) and the HR for a 10 mg daily dose of macitentan was 0.50 (95%CI, 0.34–0.75; *P* < 0.001). The efficacy of macitentan was observed regardless of whether the patient was receiving specific therapy for PAH at study entry.

In month 6, the 6 min walk distance had decreased by a mean of 9.4 m in the placebo group. In contrast, the 6 min walk distance had increased by a mean of 7.4 m in the 3 mg macitentan arm (treatment effect, 17 m; 97.5%CI, −2.7–36.4; *P* = 0.01) and by a mean of 12.5 m in the 10 mg macitentan group (treatment effect, 22 m; 97.5%CI, 3.2–40.8; *P* = 0.008). These effects were also examined according to whether or not the patient was receiving therapy for PAH and according to the WHO functional class at baseline. The functional class improved from baseline to month 6 in 13% of the patients in the placebo group, as compared with 20% of those receiving 3 mg of macitentan (*P* = 0.04) and 22% of those receiving 10 mg of macitentan (*P* = 0.006). A subset of patients participated in a hemodynamic study that included right heart catheterization at baseline and 6 months. Patients in both macitentan arms had significant decreases in pulmonary vascular resistance and increases in cardiac index.

The adverse events most commonly associated with being assigned to macitentan versus placebo were headache, nasopharyngitis, and anemia. Rates of discontinuation due to adverse events were 12% in the placebo group, 14% in the 3 mg macitentan group, and 11% in the 10 mg macitentan group. Women who are prescribed macitentan need to participate in a risk evaluation and mitigation strategy program due to the risk for fetal harm like other medications in this class, including bosentan and ambrisentan.

The strengths of SERAPHIN are (1) the largest and longest Phase III outcome trial to date on a novel pharmacological treatment for PAH; and (2) the first study powered for a hard clinical endpoint (morbidity and mortality) instead of just change in functional class or 6 min walk distance.

## 4. Phosphodiesterase Type-5 Inhibitors

The pulmonary vasculature contains sizeable amounts of phosphodiesterase type-5 [39–41]. Therefore, a strategy for increasing the activity of endogenous nitric oxide in PAH is to enhance nitric oxide—dependent, intracellular cyclic guanosine monophosphate (cGMP)—mediated pulmonary vasodilatation through inhibition of the breakdown of cGMP by phosphodiesterase type-5. All three phosphodiesterase type-5 inhibitors approved for the treatment of erectile dysfunction, sildenafil, tadalafil, and vardenafil cause significant pulmonary vasodilation, with maximum effects observed after 60, 75-90, and 40–45 min, respectively [41]. In addition, phosphodiesterase type-5 inhibition has been shown to exert also antiproliferative effects [39, 40].

Two randomized controlled trials have tested the effects of orally active phosphodiesterase type-5 inhibitors (namely, sildenafil and tadalafil) in patients with PAH.  Table 3 summarizes the characteristics of patients, etiology, end points, treatment effects, and adverse reactions in these trials.

### 4.1. Sildenafil

It was approved by the FDA in 2005. The approval of sildenafil at 20 mg three times daily for the treatment of patients with PAH was to improve exercise ability, regardless of the functional class or etiology. The approval was based on the results from SUPER-1 (Sildenafil Use in Pulmonary artERial Hypertension-1) trial [32].

This was a multicenter, double-blind, randomized study that evaluated the effects of sildenafil 20, 40, or 80 mg three times daily compared with placebo for 12 weeks in 278 adults with PAH that was idiopathic or associated with connective tissue disease or congenital shunts (repaired at least 5 years earlier). After three months of treatment, the mean placebo-adjusted changes in 6 min walk distance for 20, 40, and 80 mg doses of sildenafil were 45 m, 46 m, and 50 m, respectively (Table 3). Furthermore, significant hemodynamic and functional class improvements were noted in every sildenafil group as compared to placebo. Common side effects of treatment with sildenafil include headache, flushing, and dyspepsia.

Of 278 patients treated in SUPER-1, 257 completed the trial and entered an open-label, uncontrolled extension phase (SUPER-2) receiving the 80 mg dose [42]. After 3 years, most patients (60%) improved or maintained their functional status noted at the time of SUPER-1 entry, and 46% maintained or improved their 6 min walk distance. Three-year estimated survival was 79% and no deaths were considered to be treatment-related. Although the approved dose of sildenafil is 20 mg, in clinical practice, uptitration beyond 20 mg (mainly 40 or 80 mg) is needed quite frequently to preserve the durability of effect.

### 4.2. Tadalafil

In contrast to sildenafil, tadalafil has a long half-life (35 h), which allows once daily administration. It was granted for use by the FDA in 2009. Similar to sildenafil, its regulatory approval (at a dose of 40 mg once daily) was based on the results from a single pivotal trial, PHIRST (Pulmonary arterial HypertensIon and ReSponse to Tadalafil) [33].

This was a multicenter, double-blind, randomized study that enrolled 405 patients who were either treatment-naïve or receiving background bosentan therapy (53%). Patients were assigned to placebo or one of the several proposed doses of tadalafil (2.5, 10, 20, or 40 mg once daily) for a period of 16 weeks (Table 3). Most patients had idiopathic PAH and functional class II or III symptoms. The primary efficacy end point was a change in 6 min walk distance. Secondary end points were changed in Borg dyspnea score, WHO functional class, time to clinical worsening, quality of life (using the EuroQol and SF-36 scales), and (in a subset of patients) cardiopulmonary hemodynamics [33].

Tadalafil increased the distance walked in 6 min in a dose-dependent manner; only the 40 mg dose met the prespecified level of statistical significance (*P* < 0.01). Overall, the mean placebo-corrected treatment effect was 33 m. In the bosentan-naïve group, the treatment effect was 44 m compared with 23 m in patients on background bosentan therapy. Tadalafil 40 mg improved the time to clinical worsening (*P* = 0.04), incidence of clinical worsening (68% relative risk reduction; *P* = 0.04), and health-related quality of life. The changes in WHO functional class were not statistically significant. The most common treatment-related adverse events reported with tadalafil were headache, myalgia, back pain, and flushing. Data analysis of comparative hemodynamic data from 93 patients, who underwent repeat right heart catheterization, demonstrated improvements with tadalafil 20 and 40 mg compared to baseline in mean pulmonary arterial pressure (*P* < 0.001 and *P* = 0.01, resp.) and pulmonary vascular resistance (*P* = 0.001 and *P* = 0.04, resp.).

## 5. Guanylate-Cyclase Stimulators

Soluble guanylate-cyclase is a key enzyme in the nitric oxide signaling pathway. On binding of nitric oxide to its prosthetic heme group, soluble guanylate-cyclase catalyzes the synthesis of the second messenger cGMP, which, as mentioned in earlier text, promotes vasodilation and inhibits smooth muscle proliferation as well as leukocyte recruitment, platelet aggregation, and vascular remodeling through a number of downstream mechanisms. The central role of the nitric oxide-soluble guanylate-cyclase-cGMP pathway in regulating pulmonary vascular tone is demonstrated by the dysregulation of nitric oxide production, soluble guanylate-cyclase activity, and cGMP degradation in PAH.

The soluble guanylate-cyclase stimulators are novel pharmacological agents that directly stimulate soluble guanylate-cyclase activity independently of nitric oxide [43, 44]. They increase the sensitivity of soluble guanylate-cyclase to endogenous bioavailable nitric oxide and mimic the effects of nitric oxide when it is absent or insufficiently produced by endothelial cells [43, 44].

The clinical effects of guanylate-cyclase stimulators (namely, riociguat [licensed in October 2013 by the FDA]) have been tested in two recently published Phase III randomized controlled trials, PATENT-1 (Pulmonary Arterial hyperTENsion sGC-stimulator Trial) and CHEST-1 (CHronic thromboEmbolic pulmonary hypertension sGC-Stimulator Trial), which are summarized in Table 4 [34].

The 12-week PATENT-1, which was conducted at 124 centers in 30 countries, included 443 patients who had symptomatic PAH. Half of the patients were not receiving any treatments for PAH, 44% were taking an endothelin-receptor antagonist, and 6% were taking a nonintravenous prostanoid. The use of phosphodiesterase type-5 inhibitorsand intravenous prostanoids was not allowed.

The patients were randomized to placebo or riociguat with individually tailored doses (range 0.5–2.5 mg) three times daily for 12 weeks. Dose adjustments were made through the first 8 weeks based on systolic systemic arterial blood pressure and signs or symptoms of hypotension.

The average 6 min walk distance at the start of the study was 368 m in the placebo group and 361 m in the riociguat group. Through 12 weeks, the distance dropped by an average of 6 m with placebo and increased by an average of 30 m with riociguat.

Prespecified subgroup analyses showed that riociguat improved the 6 minute walking distance in both patients who had not received other treatment for the disease and those who had been on endothelin-receptor antagonists or prostanoids. There were significant improvements in pulmonary vascular resistance (*P* < 0.001), N-terminal probrain natriuretic peptide levels (*P* < 0.001), functional class (*P* = 0.003), time to clinical worsening (*P* = 0.005), and Borg dyspnea score (*P* = 0.002). The drug was associated with a lower rate of serious adverse events. The most common such events were headache, peripheral edema, hypotension, dizziness, and syncope.

The CHEST-1 trial was conducted at 89 centers in 26 countries and included 261 patients who had inoperable chronic thromboembolic pulmonary hypertension or recurrent pulmonary hypertension after endarterectomy and who were not taking endothelin-receptor antagonists, prostanoids, or phosphodiesterase type-5 inhibitors, within 3 months of the start of the study [35].

The patients were randomized to either placebo or riociguat given at an adjusted dose up to 2.5 mg three times daily for 16 weeks. At baseline, the average 6 min walk distance was 356 m in the placebo group and 342 m in the riociguat group. By the end of the study, the average distance decreased by 6 m in the placebo group and increased by 39 m in the riociguat group.

Secondary end points included changes from baseline in pulmonary vascular resistance, N-terminal probrain natriuretic peptide level, WHO functional class, time to clinical worsening, Borg dyspnea score, quality of life variables, and safety.

Pulmonary vascular resistance decreased by 226 dyne/s/cm^−5^ in the riociguat arm and increased by 23 dyne/s/cm^−5^ in the placebo group (*P* < 0.001). Riociguat was also associated with significant improvements in the N-terminal probrain natriuretic peptide level (*P* < 0.001) and functional class (*P* = 0.003).

The most common serious adverse events were right ventricular failure (in 3% of patients in each arm) and syncope (in 2% of the riociguat arm and 3% of the placebo arm). The rate of discontinuation due to adverse events was 3% with riociguat and 2% with placebo. There was a greater percentage of deaths in the placebo arm (3%* versus* 1%), although one death from acute renal failure in the riociguat arm was considered to be related to the study treatment.

Of note, in open-label extension studies (PATENT-2 and CHEST-2), riociguat patients exhibited further increases in 6 min walk distance by 10–20 m over 12 weeks of additional treatment; these were then maintained for an entire year of therapy.

## 6. Combination of FDA Approved Drugs

Since the existing PAH therapies act by different, yet potentially complementary mechanisms, using combination pharmacotherapy may provide additive and/or synergistic effects by simultaneously addressing multiple disease pathways. Another potential advantage of using combined drugs is reduced or minimized side effects/toxicity by using lower doses.

A few randomized controlled trials have been published on combination pharmacotherapy for PAH.

The BREATHE-2 was the first randomized, double-blind, placebo-controlled trial to explore the potential clinical benefits of combining oral bosentan with epoprostenol in 33 patients with severe PAH (WHO functional class III/IV) [45]. Reduction in pulmonary vascular resistance was greater with combination therapy, although it did not reach statistical significance. Furthermore, no benefit could be shown on the 6 min walk distance with combination therapy compared to epoprostenol alone: the median 6 min walk distances at week 16 in the epoprostenol + bosentan and epoprostenol + placebo groups were 68 m* versus* 74 m, respectively (*P* = NS). Of note, these results may be related to the addition of a treatment to a drug (epoprostenol) known to induce significant clinical benefits in severe PAH patients.

The subsequent randomized controlled STEP (Safety and Pilot Efficacy Trial of Inhaled Iloprost in Combination with Bosentan for Evaluation in Pulmonary Arterial Hypertension) addressed the safety and efficacy of 12-week therapy with inhaled iloprost in 67 patients with idiopathic PAH or associated PAH in NYHA functional class III already treated with bosentan [46]. The investigators found a significant increase in the 6 min walk distance from baseline to postinhalation measurements at week 12 in the bosentan + iloprost arm versus placebo (30 m [*P* = 0.001] and 4 m [*P* = 0.69], resp.). The preinhalation 6 min walk distance change from baseline at week 12 was 29 m (*P* = 0.007) and 11 m (*P* = 0.45) in the bosentan + iloprost and placebo groups, respectively. However, significance was lost with a placebo-adjusted difference for both pre- and postinhalation measurements. Changes in hemodynamic parameters between the two study groups were found to be only statistically significant in mean pulmonary arterial pressure from baseline to postinhalation with a placebo-adjusted change of −8 mmHg. The study also showed significant improvement in New York Heart Association functional class (34% versus 6%, resp.; *P* = 0.002) and time to clinical deterioration (0% versus 15%, resp.; *P* = 0.02). Combination therapy proved both efficacious and well tolerated; syncope was notably less frequent and severe in the combination treatment arm than that reported in the AIR trial, possibly because of background bosentan therapy.

The PACES (Pulmonary Arterial Hypertension Combination Study of Epoprostenol and Sildenafil) trial tested the sequential addition of oral sildenafil 80 mg three times daily in 267 PAH patients already receiving intravenous epoprostenol with insufficient clinical improvement (mean duration was ~3 years before study entry) [47]. The most relevant findings of this study were significant improvements after 16 weeks in 6 min walk distance (+26 m, *P* < 0.001) and hemodynamic parameters. Of note, there was a significant reduction in the number of patients showing clinical worsening (defined as death, lung transplantation, hospitalization secondary to PAH, bosentan therapy initiation, or epoprostenol dose change by > 10% because of clinical deterioration) and an improvement of survival with seven deaths occurring all in the placebo arm. The adverse events of sildenafil were similar to placebo suggesting that most patients were able to tolerate the higher doses.

Besides the above randomized trials, several uncontrolled studies assessed the efficacy and safety of combination therapy with promising results. The open label sequential addition of bosentan or sildenafil to treprostinil or iloprost was shown to have additive beneficial effects and be safe [48–50]. Also, the addition of tadalafil or sildenafil to bosentan monotherapy, both available in oral form, improved New York Heart Association functional class and 6 min walk distance and offered an exciting option with the possibility of avoiding more complex parenteral therapies [51, 52].

However, two recent meta-analyses have mined the effects of combination pharmacotherapy [53, 54]. Zhu and colleagues have reported an increase in exercise capacity and a reduced risk of clinical worsening with combination therapy compared to monotherapy in seven PAH randomized controlled trials, whereas Fox and colleagues, out of six randomized controlled trials, reported that in PAH combination therapy does not offer an advantage over monotherapy for preventing clinical worsening (death, hospital admission, transplantation, and treatment escalation) apart from modestly increasing exercise capacity [53, 54].

Nonetheless, combination pharmacotherapy has become the standard of care in manycenters even if its long-term safety and efficacy have not yet been fully assessed. Additionally, the current limited data precludes consensus on which agents to combine, when to switch, when to combine, and the optimal timing (initial combination [in naïve patients], sequential combination[in case of clinical deterioration with the first drug], or when the therapeutic goals are not met [goal-oriented therapy]).

Current US and European guidelines for the treatment of PAH have given a grade IIA to IIB recommendation for combination therapy in PAH (interpretation-weight of evidence/opinion is in favor of usefulness/efficacy) [5, 55]. They propose sequential combination therapy for more severe cases that fail to respond to monotherapy. Moreover, it is recommended that combination therapy takes place under the supervision of experienced PAH-specialty practitioners or within the context of clinical trials or registries.

Therefore, further research with randomized controlled trials should be performed to provide more consistent information before establishing final guidelines. As suggested by Galiè and colleagues, the appropriate design to assess the efficacy of this strategy appears to be a three-arm study, comparing combination therapy with two arms of monotherapy using the single agents [56].

## 7. Expected Novel Drugs and Future Therapeutic Targets

Despite the contemporary availability of a number of approved prostanoids, endothelin receptor antagonists, phosphodiesterase type-5 inhibitors, and guanylate-cyclase stimulators for PAH, these treatment options continue to have substantial limitations: they are neither universally available nor always effective or curative and the long-term prognosis, although improved over the past two decades, remains unfortunate.

Although vasoconstriction is a main component of the pathophysiology of PAH, particularly early in the disease, there has been a growing curiosity in the past few years not only for agents which act as vasodilators, but also for those with antiproliferative and antiremodeling effects. There is a remarkable evidence that the pulmonary vasculopathy is related to inflammation and a proproliferative/antiapoptotic phenotype of pulmonary artery smooth muscle cells.

Of note, cancer hallmarks have been found in PAH, therefore opening the way to new therapeutic strategies developed in oncology [57].

### 7.1. Selexipag

It is a long-acting orally prodrug metabolized to the prostaglandin I_2_-receptor (IP-receptor) agonist, which has a half-life of ~8 hours. In contrast with other prostanoids, selexipag is highly selective (at least 130-fold selectivity) for human IP-receptors, whereas other prostanoids can activate other prostaglandin receptors, such as E_3_ receptor [58, 59]. This allows for a greater vasodilatory effect than iloprost with less adverse reactions, which may further warrant selexipag's use at high doses.

Results of a Phase II, 43-patient, placebo-controlled, double-blind study, where patients were randomized in a 3 : 1 ratio receiving selexipag (200 *μ*g–800 *μ*g twice-daily) or placebo on background therapy with an endothelin-receptor antagonist or phosphodiesterase type-5 inhibitor or both, showed a statistically significant reduction in pulmonary vascular resistance (primary end point) [60]. The treatment effect was shown to be 30% after 17 weeks of treatment (*P* = 0.004). Results also showed an encouraging numerical improvement in 6 min walk distance, which was a secondary end point of this trial. Selexipag was well tolerated and the safety profile was in line with the expected pharmacologic effect. The most commonly reported adverse events were headache, jaw pain, leg pain, nausea, and diarrhea.

Selexipag is being evaluated in the Phase III GRIPHON, (prostacyclin [PGI_2_] Receptor agonIst in Pulmonary arterial HypertensiON) trial. This is a multicenter, double-blind, placebo-controlled trial evaluating the efficacy and safety of selexipag in patients with PAH [61]. This pivotal study, designed to demonstrate a reduction in risk of morbidity/mortality events, is fully enrolled with 1,156 patients (the largest PAH trial so far). In May 2013, an independent data monitoring committee conducted an interim analysis resulting in an unanimous recommendation to continue the study. Final study results of this event-driven study are now expected by late 2014.

### 7.2. Imatinib

This drug inhibits the tyrosine kinase activity of the Bcr-Abl oncoprotein, the stem cell factor c-Kit, and the platelet-derived growth factor receptor (PDGF-R) kinases [62–66]. Imatinib is approved for the treatment of chronic myelogenous leukemia, gastrointestinal stromal tumors, and other malignancies [62–66]. In addition to prostaglandin I_2_-, endothelin-1-, and nitric oxide-dependent pathways, the overexpression of PDGF-R has been reported to play a crucial role in the pathobiology of PAH [67, 68]. The PDGF has been implicated in endothelial cell dysfunction and proliferation and migration of smooth muscle cells [67, 68]. Pulmonary vascular remodeling in different animal models of PAH was shown to regress with the administration of imatinib [68, 69].

A randomized, double-blind, placebo-controlled Phase II study in 59 PAH patients in functional class II to IV receiving specific PAH therapies reported that imatinib significantly improved pulmonary hemodynamics [70]. In that study, a* post hoc *subgroup analysis suggested that patients with greater hemodynamic impairment might respond better to imatinib than patients with less advanced disease. This preliminary study led to the development of IMPRES (IMatinib in Pulmonary arterial hypertension, a Randomized Efficacy Study), a randomized, double-blind, placebo-controlled 24-week trial [71]. This study evaluated imatinib in patients with pulmonary vascular resistance ≥800 dynes·sec·cm^−5^ symptomatic on ≥2 PAH therapies. The primary outcome was a change in 6-min walk distance. Secondary outcomes included changes in hemodynamics, functional class, serum levels of N-terminal brain natriuretic peptide, and time to clinical worsening. After completion of the core study, patients could enter an open-label long-term extension study. After 24 weeks, the mean placebo-corrected treatment-effect on 6-min walk distance was 32 m (*P* = 0.002), an effect maintained in the extension study. Pulmonary vascular resistance decreased by 379 dynes·sec·cm^−5^ (*P* < 0.001; between-group difference). Functional class, time to clinical worsening, and mortality did not differ between treatments. Serious adverse events and discontinuations were more frequent with imatinib than placebo (44%* versus* 30%, 33%* versus* 18%, resp.). Subdural hematoma occurred in 8 patients (2 in the core study, 6 in the extension) receiving imatinib and anticoagulation. According to these results, the benefit/risk ratio of imatinib was not considered to be sufficient and to date the off-label use of imatinib for PAH is not recommended.

Nilotinib is a new generation of oral tyrosine kinase inhibitor used for the treatment of gastrointestinal stromal tumors and for newly diagnosed chronic myeloid leukemia or for patients presenting resistance or intolerance to imatinib [72]. In addition, it also has 20–50 times greater inhibitory activity against the PDGF-R and c-Kit kinases than imatinib [73]. In a monocrotaline rat model of pulmonary hypertension, nilotinib reduced right ventricular pressure and percentage of muscularized lung vessels with efficacy similar to that of imatinib [74]. A 24-week, randomized, placebo-controlled, dose ranging safety and efficacy study of nilotinib in patients with PAH is in progress [75].

### 7.3. Rho-Kinase Inhibitors

A wide variety of cellular actions, including proliferation, apoptosis, motility, migration, inflammation, and vasoconstriction are influenced and regulated by the Rho-A/Rho-kinase signaling pathway [76–79]. All these effects are related to its activation (which can occur in response to many ligands implicated in the pathogenesis of PAH, including endothelin-1, thromboxane-A_2_, and serotonin) and subsequent inhibition of the myosin light chain phosphatase activity [76–79].

Accruing evidence from a number of studies intensely suggests that Rho-A/Rho-kinase signaling plays a crucial role in the pathogenesis of various animal models of pulmonary hypertension and its activity is increased as well in the pulmonary artery smooth muscle cells of idiopathic PAH patients [79, 80].

Fasudil is a potent inhibitor of Rho-kinase, with its inhibitory effect being 100 times and 1000 times greater than on protein kinase C and myosin light chain kinase, respectively [81]. It can acutely attenuate pulmonary vascular resistance in spontaneously hypertensive fawn-hooded rats, monocrotaline-induced pulmonary hypertension in rats, and hypoxia-induced pulmonary hypertension in mice [81]. Besides its vasodilator effect, it also suppresses cellular proliferation and enhances apoptosis in monocrotaline-induced pulmonary hypertension rat models.

Of note, a recent study showed that intravenous fasudil exerts acute pulmonary vasodilator effects in patients with severe PAH who were refractory to conventional therapies [82]. Also, some clinical trials suggest that inhalation of fasudil is as effective as inhaled nitric oxide in decreasing mean pulmonary arterial pressure and pulmonary vascular resistance not only in patients with idiopathic PAH, but also in those with forms of PAH associated with connective tissue disease, congenital heart disease, or portal hypertension [83, 84].

Recently, the new highly selective Rho-kinase inhibitor azaindole-1 has demonstrated efficacy in hypoxia-and monocrotalin-induced pulmonary hypertension and may represent a further development of fasudil for the treatment of PAH [85].

### 7.4. Vasoactive Intestinal Peptide

It is a neuropeptide in the glucagon growth hormone-releasing factor secretion superfamily with a wide range of effects, including anti-inflammatory and immune-modulatory roles as well as vasodilation of the pulmonary vasculature and inhibition of pulmonary artery smooth muscle cell proliferation [86–88]. Recently, it was shown that male mice lacking the gene for vasoactive intestinal peptide spontaneously developed features of moderate-to-severe PAH [89].

In a small, prospective, controlled, intraindividual 3-month trial, administration of vasoactive intestinal peptide by inhalation (single 200-*μ*g dose) to patients with PAH has shown a significant decrease in mean pulmonary arterial pressure, pulmonary vascular resistance, and dyspnea score and increased cardiac output, mixed venous oxygen saturation, and 6 min walk distance [90]. Moreover, in an open-label study, a total of 20 patients with pulmonary hypertension (PAH in nine, secondary to lung disease in eight, and chronic thromboembolic in three) inhaled a single 100-*μ*g dose of vasoactive intestinal peptide during right-heart catheterization, which caused significant selective pulmonary vasodilation with improved stroke volume and mixed venous oxygen saturation [91]. Overall, six patients experienced a pulmonary vascular resistance reduction of >20%. Vasoactive intestinal peptide did not cause any side-effects and led to a reduced workload of the right ventricle without affecting systemic blood pressure. Further studies are needed to evaluate the full therapeutic potential of vasoactive intestinal peptide, including higher doses and chronic treatment [92].

### 7.5. Endothelial Progenitor Cells Transplantation

Endothelial dysfunction is a prominent component of PAH pathobiology. Thus, pulmonary endothelial cells may be a therapeutic target for its treatment. The endothelial progenitor cells are a cell population that has the capacity to circulate, proliferate, and differentiate into mature endothelial cells, but they have neither acquired characteristic mature endothelial markers nor formed a lumen [93–96]. These cells express some cell surface markers or transcription factors that characterize mature endothelium. Laboratory evidence suggests that these precursors participate in postnatal neovascularization and reendothelialization.

A prospective, randomized trial of 31 patients with idiopathic PAH (15 receiving autologous endothelial progenitor cells, 16 receiving conventional therapy) showed that infusion of autologous endothelial progenitor cells after 12 weeks of follow-up was associated with a significant increase in 6 min walk distance as compared with conventional therapy group [97]. The mean difference between the 2 groups was 42.5 m (95%CI: 28.7–56.3 m, *P* < 0.001). The patients in the cell infusion group also had significant improvement in mean pulmonary artery pressure, pulmonary vascular resistance, and cardiac output. There were no severe adverse events with cell infusion.

A Phase I clinical trial, PHACeT (Pulmonary Hypertension Assessment of Cell Therapy) is currently under way to confirm tolerability, safety, and efficacy of autologous endothelial progenitor cells programmed to overexpress the endothelial nitric oxide synthase [98]. Eighteen patients with idiopathic, heritable, or anorexigen-associated PAH have been enrolled in this dose-escalation study, which will evaluate long-term safety (5 years) and short-term efficacy (exercise capacity and hemodynamics at 3 months) as the main outcome measures (http://clinicaltrials.gov identifier NCT00469027).

## 8. Interventional Strategies

### 8.1. Percutaneous Balloon Atrioseptostomy

It has been established as palliative treatment or bridge to transplantation in patients with right ventricular failure from severe PAH. It consists of puncture of the atrial septum followed by repetitive balloon septal dilatation.

Atrioseptostomy aims at creating a safety valve by unloading the right heart and increasing left ventricular preload and output, peripheral perfusion, net oxygen tissue delivery, and exercise tolerance. However, the periprocedural mortality rate is relatively high due to the critical hemodynamics of these patients and the risk of aortic/cardiac perforation and tamponade during transseptal puncture by using the traditional approach with Brockenbrough's needle [99, 100].

In recent years, novel approaches to atrioseptostomy, using either radiofrequency perforation, cutting balloon dilatation, or insertion of butterfly stents under intracardiac echocardiographic guidance, have been proposed [101–103]. These new techniques are feasible and associated with very low rate of major complications and death.

With the availability of these new interventional strategies, we believe that balloon atrioseptostomy may be considered at an earlier stage rather than late in the progression of PAH; when all available medical therapy fails, the risk of procedural death is increased and the impact of the intervention on long-term survival is minimized.

In a relatively large series of patients with PAH, the early sequential combination of balloon atrioseptostomy before PAH-specific pharmacological therapy appeared to exert a beneficial impact on long-term survival, which was superior to that provided by atrioseptostomy alone [104]. Fifty procedures performed in 34 patients resulted in haemodynamic and symptomatic improvement in most of the patients. Only one (2%) procedure-related death occurred. In 21 patients, atrioseptostomy was the only form of treatment, while 11 received additional pharmacotherapy after atrioseptostomy. During follow-up (58.5 ± 38 months), 21 patients died; median survival of the group was 60 months (95%CI 43–77 months). Median survival for patients on pharmacotherapy additional to atrioseptostomy was significantly longer than that for patients receiving atrioseptostomy alone (83 months [95%CI 57–109] versus 53 months [95%CI 39–67]; log-rank 6.52; *P* = 0.01).

### 8.2. Percutaneous Pulmonary Artery Denervation

Several experimental studies showed that pulmonary hypertension may be secondary to the distention of the main pulmonary artery by the excitation of stretch receptors in or near the bifurcation area of the main pulmonary artery [12–17]. Distention and occlusion of one branch of the pulmonary artery by the use of balloons and nonocclusive inflatable cuffed cylinders led to significant increases of pulmonary arterial pressure and pulmonary vascular resistance [12–17].

Juratsch et al. reported that the significantly increased pulmonary arterial pressure and pulmonary vascular resistance induced by balloon distension of the main pulmonary artery were completely abolished by surgical denervation of the bifurcation of the main pulmonary artery and chemical sympathectomy using 6-hydroxydopamine (a known mediator of adrenergic nerves) [13]. These results suggested that the efferent limb of this reflex is predominantly mediated via the adrenergic nervous system.

Recently, the first-in-man PADN-1 (**P**ulmonary** A**rtery** D**e**N**ervation for treatment of pulmonary arterial hypertension) study, by using a dedicated 7.5-F multiple-function (temperature sensor and radiofrequency ablation) catheter positioned through a peripheral vein (patent application in progress) has been published [105].

Chen et al. studied 21 patients with idiopathic PAH who were treated with either radiofrequency catheter-based pulmonary artery denervation alone (*n* = 13) or continued medical therapy (*n* = 8). Average time interval between the onsets of symptoms to diagnosis was 3.5 years. All patients did not respond optimally to current medical treatment after an average of 3.3 years of therapy using at least 2 drugs. Pulmonary denervation was performed at the bifurcation of the main pulmonary artery and at the ostial right and left pulmonary artery. Serial echocardiography, right heart catheterization, and a 6 min walk distance test were performed.

The primary end points were the change of pulmonary artery pressure, tricuspid excursion (Tei) index, and 6 min walk distance at 3-month follow-up. Compared with the control group, at 3-month follow-up, the patients who underwent pulmonary denervation showed significant reduction of mean pulmonary arterial pressure (from 55 ± 5 mmHg to 36 ± 5 mmHg, *P* < 0.01), significant improvement in 6 min walk distance (from 324 ± 21 m to 491 ± 38 m, *P* < 0.006), and the Tei index (from 0.3 ± 0.04 to 0.50 ± 0.04, *P* < 0.001). Following the decrease in pulmonary arterial pressure, cardiac output increased from 2.0 ± 0.2 L/min/m^2^ to 2.8 ± 0.3 L/min/m^2^ (*P* < 0.001) with associated reduction of transpulmonary pressure gradient and pulmonary vessel resistance and increase in venous oxygen saturation in the pulmonary artery.

Procedural success was 92.3%, with one patient experiencing intolerable chest pain induced by denervation. Rehospitalization was needed in more than half (62.5%) of controls and none of the denervation patients (*P* < 0.001). Two patients in the study arm died at 3 months, one from septic shock and the other from right ventricular failure. All medications were discontinued safely apart from one patient who continued receiving a diuretic after denervation. This might imply the “pure” effect of denervation itself on improvements in cardiac function, hemodynamic measurements, and functional capacity. However, as pointed out by Galiè and Manes, this study should be considered as a very preliminary proof-of-principle study that requires a formal and large multicenter randomized controlled trial to appropriately evaluate a possible new era for the treatment of PAH patients [106].

## 9. Conclusions

There have been substantial progresses in the last five years in the understanding of the pathobiology and pathophysiology of PAH, which led to the developments of new pharmacological and interventional strategies. New oral forms of prostanoids (treprostinil) and novel endothelin-receptor antagonists (macitentan) and guanylate-cyclase stimulators (riociguat) have transformed the prognosis for PAH patients from symptomatic improvements in exercise tolerance ten years ago to delayed disease progression today offering an exciting option with the possibility of avoiding more complex parenteral therapies. On the other hand, percutaneous balloon atrioseptostomy by using radiofrequency perforation, cutting balloon dilatation, or insertion of butterfly stents and pulmonary artery catheter-based denervation, both associated with very low rate of major periprocedural complications and death, should be considered in combination with specific drugs at an earlier stage rather than late in the progression of PAH and before the occurrence of overt right-sided heart failure.

## Figures and Tables

**Table 1:** Patients, etiology, end points, treatment effects, and adverse reactions in the Pivotal Phase III Randomized Controlled Trials of the US Food and Drug Administration approved prostanoids for treatment of pulmonary arterial hypertension in adults.

	Rubin et al. [20]	Barst et al. [21]	Badesch et al. [22]	AIR [23]	Simmoneau et al. [24]	TRIUMPH-1 [25]	FREEDOM-M [26]
Patients (no.)	23	81	111	203	470	235	349
Drug	*EPOPROSTENOL *			*ILOPROST *	*TREPROSTINIL *		
Dosing/route	20–40 ng/Kg/min iv.			2.5–5 *μ*g inh. x6–9	20–80 ng/Kg/min sc.	18–54 *μ*g inh. x4	0.125–12 mg bid os
Follow-up (months)	2	3	3	3	3	3	3
Etiology (%)*							
IPAH	100	100	—	51	58	56	74
CTD	—	—	100	13	19	33	18
CHD	—	—	—	—	24	—	7
CTEPH	—	—	—	33	—	—	—
Anorexigen use	—	—	—	3	—	—	—
HIV	—	—	—	—	—	—	1
Functional class (%)							
NYHA/WHO II	9	—	5	—	11	—	34
NYHA/WHO III	65	75	78	59	82	98	66
NYHA/WHO IV	26	25	17	41	7	2	—
Primary end point	Hemodynamics	Hemodynamics	6MWD	Combined^†^	6MWD	6MWD	6MWD
Treatment effects							
Δ6MWD (m)	45	47	94	36	16	20	23
Hemodynamics	Improved	Improved	Improved	Improved	Improved	N/A	N/A
Clinical worsening	Reduced	Reduced^‡^	No change	Reduced	Reduced	No change	No change
Adverse reactions	Flushing, headache, vomiting, jaw pain, hypotension			Flushing, cough, headache, trismus	Infusion site pain, cough, headache	Pharyngeal pain/throat irritation, cough	Nausea, diarrhea, headache, jaw pain

AIR: Aerosolized Iloprost Randomized; TRIUPMH: TReprostinil Sodium Inhalation Used in the Management of Pulmonary Arterial Hypertension; FREEDOM-M: Oral Treprostinil as Monotherapy for the Treatment of Pulmonary Arterial Hypertension; iv.: intravenous; inh.: inhalation; sc.: subcutaneous; bid: twice daily; os: oral; *sum of percentage may not be 100% for rounding to the nearest unit; 0.5 is rounded to the upper unit; IPAH: idiopathic pulmonary arterial hypertension; CTD: connective tissue disease; CHD: congenital heart disease (congenital systemic-to-pulmonary shunts); CTEPH: chronic thromboembolic pulmonary hypertension; HIV: human immunodeficiency virus; NYHA: New York Heart Association; WHO: World Health Organization; 6MWD: 6 min walk distance; ^†^combined epoprostenol end point of improvement in NYHA functional class and >10% in 6MWD; Δ: mean (or median) change from baseline;^‡^ significantsurvival benefit (*P* = 0.003) was observed in epoprostenol patients.

**Table 2:** Patients, etiology, end points, treatment effects, and adverse reactions in the Pivotal Phase III randomized controlled trials of the US Food and Drug Administration approved endothelin-1 receptor antagonists for the treatment of pulmonary arterial hypertension in adults.

	Channick et al. [27]	BREATHE-1 [28]	EARLY [29]	ARIES-1 [30]	ARIES-2 [30]	SERAPHIN [31]
Patients (no.)	32	213	185	202	192	742
Drug	*BOSENTAN *			*AMBRISENTAN *		*MACITENTAN *
Dosing/route	62.5–125 mg bid/os	62.5–250 mg bid/os	62.5–125 mg bid/os	5 or 10 mg qd/os	2.5 or 5 mg qd/os	3 or 10 mg qd/os
Follow-up (months)	3	4	6	3	3	24
Etiology (%)*						
IPAH	85	70	58	63	65	56
CTD	15	30	20	29	30	30
CHD	—	—	17	—	—	8
HIV	—	—	5	5	3	1
Anorexigen use	—	—	—	3	2	3
Functional class (%)						
NYHA/WHO II	—	—	100	30	45	52
NYHA/WHO III	100	91	—	60	52	46
NYHA/WHO IV		9	—	10	3	2
Primary end point	6MWD	6MWD	6MWD Hemodynamics	6MWD	6MWD	Composite/events^†^
Treatment effects						
Δ6MWD (m)	76	44	19	31 and 51	32 and 59	17 and 22
Hemodynamics	Improved	N/A	Improved	No change	No change	Improved
Clinical worsening	Reduced	Reduced	Reduced	Reduced	Reduced	Reduced
Adverse reactions	Headache, flushing, syncope, anemia, and elevated liver aminotransferase values			Peripheral edema, nasal congestion, and flushing		Anemia, headache, and nasopharyngitis

BREATHE: Bosentan Randomized Trial of Endothelin Antagonist THErapy; EARLY: Endothelin Antagonist tRial in mildLY symptomatic pulmonary arterial hypertension patients; ARIES: Ambrisentan in pulmonary arterial hypertension, RandomIzed, double-blind, placebo-controlled, multicenter, efficacy study; SERAPHIN: Study with an Endothelin Receptor Antagonist in Pulmonary Arterial Hypertension to Improve cliNical outcome; bid: twice daily; qd: once-daily; os: oral; *sum of percentage may not be 100% for rounding to the nearest unit; 0.5 is rounded to the upper unit; IPAH: idiopathic pulmonary arterial hypertension; CTD: connective tissue disease; CHD: congenital heart disease (congenital systemic-to-pulmonary shunts); HIV: human immunodeficiency virus; NYHA: New York Heart Association; WHO: World Health Organization; 6MWD: 6 min walking distance; ^†^primary end point, time from the initiation of treatment to the first occurrence of a composite of death, atrioseptostomy, lung transplantation, initiation of prostanoids, or worsening of PAH; Δ: mean (or median) change from baseline.

**Table 3:** Patients, etiology, end points, treatment effects, and adverse reactions of the US Food and Drug Administration approved phosphodiesterase type-5 inhibitors in the pivotal Phase III randomized controlled trials for treatment of pulmonary arterial hypertension in adults.

	SUPER-1 [32]	PHIRST [33]
Patients (no.)	278	405
Drug	*Sildenafil *	*Tadalafil *
Dosing/route	20, 40 or 80 mg tid/os	2.5, 10, 20, or 40 mg qd/os
Follow-up (months)	3	4
Etiology (%)*		
IPAH	64	60
CTD	30	24
CHD	6	11
Anorexigen use	—	4
Functional class		
NYHA/WHO II	36	34
NYHA/WHO III	61	62
NYHA/WHO IV	3	2
Primary end point	6MWD	6MWD
Treatment effects		
Δ6MWD (m)	45, 46, and 50	14, 20, 27 and 33^†^
Hemodynamics	Improved	Improved^¶^
Clinical worsening	Reduced	Reduced^#^
Adverse reactions	Epistaxis, headache, dyspepsia, flushing	Headache, myalgia, back pain, flushing

SUPER: Sildenafil Use in Pulmonary artERial hypertension; PHIRST: Pulmonary Arterial HypertensIon and ReSponse to Tadalafil; tid: three times daily; os: oral; qd: once-daily; *sum of percentage may not be 100% for rounding to the nearest unit; 0.5 is rounded to the upper unit; IPAH: idiopathic pulmonary arterial hypertension; CTD: connective tissue disease; CHD: congenital heart disease (systemic-to-pulmonary shunts); NYHA: New York Heart Association; WHO: World Health Organization; 6MWD: 6 min walk distance; Δ: mean (or median) change from baseline; ^†^only the 40 mg dose met the prespecified level of statistical significance (*P* < 0.01); ^¶^improvements were observed only with 20 and 40 mg doses in mean pulmonary arterial pressure and pulmonary vascular resistance; ^#^only the 40 mg dose improved the time to clinical worsening, incidence of clinical worsening, and quality of life.

**Table 4:** Patients, etiology, end points, treatment effects, and adverse reactions of the US Food and Drug Administration approved soluble guanylate cyclase stimulators in the pivotal Phase III randomized controlled trials for treatment of pulmonary arterial hypertension in adults.

	PATENT-1 [34]	CHEST-1 [35]
Patients (no.)	443	261
Drug	*RIOCIGUAT *	
Dosing/route	0.5–2.5 mg tid/os	
Follow-up (months)	3	4
Etiology (%)*		
IPAH	63	—
CTD	25	—
CHD	8	—
Portopulmonary	3	—
Anorexigen use	1	—
CTEPH		
Inoperable	—	70
Postoperative	—	30
Functional class		
NYHA/WHO I	3	2
NYHA/WHO II	40	32
NYHA/WHO III	56	62
NYHA/WHO IV	1	5
Primary end point	6MWD	6MWD
Treatment effects		
Δ6MWD (m)	30	39
Hemodynamics	Improved	Improved
Clinical worsening	Reduced	No change
Adverse reactions	Headache, peripheral edema, hypotension, dizziness, and syncope	

PATENT: Pulmonary Arterial hyperTENsion Soluble Guanylate-Cyclase-Stimulator Trial; CHEST: CHronic thromboEmbolic Pulmonary Hypertension Soluble Guanylate-Cyclase-Stimulator Trial; tid: three times daily; os: oral; *sum of percentage may not be 100% for rounding to the nearest unit; 0.5 is rounded to the upper unit; IPAH: idiopathic pulmonary arterial hypertension; CTD: connective tissue disease; CHD: congenital heart disease (congenital systemic-to-pulmonary shunts); CTPEH: chronic thromboembolic pulmonary hypertension; NYHA: New York Heart Association; WHO: World Health Organization; 6MWD: 6 min walk distance; Δ: mean (or median) change from baseline.
